# Ultrasound Guided Biopsy among Patients with Lung Lesions Undergoing Procedures in Interventional Radiology of a Tertiary Care Centre

**DOI:** 10.31729/jnma.8397

**Published:** 2024-01-31

**Authors:** Prakash Kayastha, Binaya Adhikari, Sharma Paudel, Sundar Suwal, Shashi Shekhar Shingh, Pradip Chapagain, Pradeep Raj Regmi

**Affiliations:** 1 Department of Radiology and Imaging, Tribhuvan University Teaching Hospital, Maharajgunj, Kathmandu, Nepal

**Keywords:** *biopsy*, *interventional radiology*, *lung neoplasms*, *prevalence*

## Abstract

**Introduction:**

Minimally invasive image-guided percutaneous core needle biopsy can obtain tissue samples for diagnosis of subpleural lung cancer, which is crucial for the correct management of lung lesions. Common complications of lung biopsy include pneumothorax, parenchymal haemorrhage and haemoptysis. The study aimed to determine the prevalence of ultrasound-guided biopsy among patients with lung lesions undergoing procedures in interventional radiology of a tertiary care centre.

**Methods:**

A descriptive cross-sectional study was performed in the Department of Radiology and Imaging from 1 August 2018 to 30 September 2019 after obtaining ethical approval from the Institutional Review Committee. USG-guided biopsy of peripheral lung lesions was performed with an 18 gauge semiautomatic biopsy instrument and a 17 gauge coaxial needle. A convenience sampling method was used. The point estimate was calculated at a 95% Confidence Interval.

**Results:**

Among 188 biopsy of lung lesions, ultrasound-guided biopsies were performed in 28 (14.89%) (9.80-19.98, 95% Confidence Interval).

**Conclusions:**

The prevalence of ultrasound guided biopsy among lung lesions is lower than other studies done in similar settings.

## INTRODUCTION

Imaging offers an accurate method of localizing the lung lesions from the skin and provides relevant information on needle trajectory within the lung.^1^ Commonly described complication of lung biopsy is pneumothorax and haemorrhage.^2^ Lung cancer is the most common cancer worldwide with an incidence of 16.54% in Nepal.^3,4^ Ultrasonography (USG) is an easily accessible modality in rural places of Nepal compared to computed tomography and it is easier to localize the peripheral lung lesion in ultrasound and biopsy the lesion for accurate diagnosis leading to early treatment thereby reducing mortality and morbidity.^3^

Risk of various complications is affected by different parameters such as biopsy needle size, lesion size, lesion depth from pleura, and number of pleural punctures during biopsy.^3^ There are very few literature regarding image-guided lung biopsy in our setting.

The study aimed to determine the prevalence of ultrasound-guided biopsy among patients with lung lesions undergoing procedures in interventional radiology of a tertiary care centre.

## METHODS

A descriptive cross-sectional study was conducted among patients referred to the Department of Radiology and Imaging of Tribhuvan University Teaching Hospital for a biopsy of suspicious subpleural lung lesions after obtaining ethical approval from the Institutional Review Committee of the Institute of Medicine (Reference number: 22(6-11-E)^2^/075/076). Data was collected from 1 August 2018 to 30 September 2019. Patients suspected of lung cancer sent for biopsy of lung lesions were included in the study. Patients with abnormal coagulation parameters, lung lesion size of less than 1 cm and those who did not give consent were excluded from the study. Convenience sampling was used. The sample size of the study was calculated using the formula:


$$\begin{array}{ll} \text{n} & ={\text{Z}}^{2}\times \frac{\text{p}\times \text{q}}{{\text{e}}^{\text{2}}}\\ & =1.96^{2}\times \frac{0.50\times 0.50}{0.08^{2}}\\ & =151 \end{array}$$

Where,

n = minimum required sample size
Z = 1.96 at 95% Confidence Interval (CI)
p = prevalence taken as 50% for maximum sample calculation
q = 1-p
e = margin of error, 8%

The calculated sample size was 151. However, we took 188 samples for the study.

An ultrasound-guided core needle biopsy of a subpleural lung lesion was obtained using an 18G semiautomatic biopsy instrument and a 17G coaxial needle. Demographic information of the subject, size and location of the lesion, distance of the lesion from skin and hilum, emphysema in traversing lung parenchyma, and complications following the biopsy were recorded. Histopathology reports of the biopsied specimens were traced and recorded as well.

Data were entered and analysis was performed using Microsoft Excel 2019. The point estimate was calculated at a 95% CI.

## RESULTS

Among 188 biopsies for lung lesions, ultrasound- guided biopsies were performed in 28 (14.89%) (9.8019.98, 95% CI). Among them 18 (64.29%) were male and 10 (35.71%) were female. The age range was 27-86 years with a mean age of 61.14±16.68 years.

Pneumothorax being the most common complication was seen in 3 (10.71%) patients. Only 1 (3.57%) patient had complications of hemoptysis and 1 (3.57%) had complication of parenchymal haemorrhage (Table 1).

**Table 1:** Characteristics of peripheral lung lesions in biopsy sample (n= 28).

Variables	n(%)
**Size (cm)**	
<2	5 (17.85)
2-4	19 (67.85)
>4	4 (14.28)
**Location in lung**	
Left upper lobe	13 (46.42)
Left lower lobe	4 (14.28)
Right upper lobe	6 (21.42)
Right middle lobe	4 (14.28)
Right lower lobe	1 (3.57)
**Depth from skin (cm)**	
<3	24 (85.71)
3-6	3 (10.71)
>6	1 (3.57)
**Distance from hilum (cm)**	
<2	1 (3.57)
2-4	13 (46.42)
>4	14 (50)
**Number of puncture**	
1	24 (85.71)
2	4 (14.29)
**Complications**	
Pneumothorax	3 (10.71)
Parenchymal haemorrhage	1 (3.57)
Haemoptysis	1 (3.57)

Histopathological diagnosis was obtained in 25 patients. Out of 25 patients, on histopathology of peripheral lung lesions 21 (84%) had malignant lung neoplasm (Table 2).

**Table 2:** Histopathological diagnosis of the peripheral lung lesions (n = 25).

Type of neoplasm	n (%)
Benign lung neoplasm	4 (16)
**Malignant lung neoplasm**	**21 (84)**
Adenocarcinoma	10 (40)
Squamous cell carcinoma	6 (24)
Spindle cell carcinoma	1 (4)
Metastasis	1 (4)
Inadequate	3 (12)

## DISCUSSION

Among 188 procedures performed in the interventional radiology unit for lung lesions, ultrasound-guided biopsies were performed in 14.89%. A study done in Canada by retrospectively reviewing hospital data showed the subplural lung and plural biopsy was done under ultrasound guidance in 27.21%.^4^ Another study done in Italy showed 37.72% of ultrasound- guided thoracic biopsies.^5^ In comparison to these studies, ultrasound-guided lung biopsies are done less frequently in our setting.

Parenchymal haemorrhage and hemoptysis were the second most common complications in the other study.^6^ In the previous study pneumothorax was the most common complication.^7^

In our study, 7.1% of the patients showed complications following the lung biopsy, with pneumothorax (10.7%) being the most common, and hemoptysis and parenchymal haemorrhage (3.6% each) being the second most common complication. None of the patients needed active intervention, thus suggesting the prevalence of only mild complications in our study. In another study the prevalence of pneumothorax was 7%.^2^ The reason could be a smaller sample size and multiple pleural punctures in our study.

Limitations of our study include a smaller sample size. due to which the results could not be generalised to a larger population. Another limitation is an inhomogeneous sample in our study as most of the lesions were highly suspected of lung cancer clinically as well as radiologically. Thus, further study with a larger and homogeneous sample is recommended for generalizing diagnostic yield and complications of USG-guided lung biopsy in the Nepalese population.

## CONCLUSIONS

The prevalence of ultrasound guided biopsy among lung lesions is lower than other studies done in similar settings. A analytical study with a larger sample is recommended to identify factors to identify the usefullness of this interventional procedure as compared to other procedure in our setting.
